# Impact of soil water regimes and partial root-zone drying in field-grown papaya in semi-arid conditions

**DOI:** 10.1038/s41598-021-90078-w

**Published:** 2021-05-20

**Authors:** Dionei Lima Santos, Eugênio Ferreira Coelho, Rubens Alves de Oliveira, Roberto Filgueiras, Márcio da Silva Alves, Weverton Pereira Rodrigues, Eliemar Campostrini, Antônio Hélder Rodrigues Sampaio, João Batista Ribeiro da Silva Reis, Fernando França da Cunha

**Affiliations:** 1 Instituto Federal de Educação, Ciência e Tecnologia do Pará, Campus Conceição do Araguaia, Avenida Couto Magalhães, Nº 1649, Setor Universitário, Conceição do Araguaia, Pará CEP: 68540-000 Brazil; 2 Embrapa Mandioca e Fruticultura, R. Embrapa, s/n, Cruz das Almas, Bahia CEP: 44380-000 Brazil; 3 Departamento de Engenharia Agrícola, Universidade Federal de Viçosa, Av. Peter Henry Rolfs, Viçosa, Minas Gerais CEP: 36570-900 Brazil; 4 Setor de Fisiologia Vegetal, LMGV, Centro de Ciências e Tecnologias Agropecuárias, Universidade Estadual do Norte Fluminense, Av. Alberto Lamego, 2000, Campos dos Goytacazes, Rio de Janeiro, CEP: 28013602 Brazil; 5 Centro de Ciências Agrárias, Naturais e Letras, Universidade Estadual da Região Tocantina do Maranhão, Avenida Brejo do Pinto, S/N, Estreito, Maranhão CEP: 65975-000 Brazil; 6 Instituto Federal de Educação, Ciência e Tecnologia Baiano, Campus Itaberaba, Rodovia BA-233, Km 04, Itaberaba, Bahia CEP: 46880-000 Brazil; 7 Instituto Federal de Educação, Ciência e Tecnologia Baiano, Campus Bom Jesus da Lapa, BR-349, S/N - Zona Rural, Bom Jesus da Lapa, Bahia CEP: 47600-000 Brazil; 8 Empresa de Pesquisa Agropecuária do Norte de Minas Gerais, Campo Experimental do Gorutuba, Rod. MGT 122, km 155, Nova Porteirinha, Minas Gerais CEP: 39525-000 Brazil

**Keywords:** Plant stress responses, Drought

## Abstract

This study aimed to evaluate in the papaya Tainung genotype, the effects of partial root-zone drying (PRD) technique on soil water regimes by using different frequencies of shifting irrigation-side of plant row and the effects of PRD technique on (1) crop agronomic performance, (2) titratable fruit acidity (TA), (3) total soluble solids (TSS), and TSS/TA ratio. Also, we analyze the spatial dynamic of papaya condition using normalized difference vegetation index (NDVI) from different satellite images. The study was conducted in the semi-arid region of Bahia (BA) and Minas Gerais (MG), Brazil. The combination of 100% (Full irrigation—FU), 50%, and 35% in the irrigation depth (WID) and frequencies of shifting plant-row side irrigation of 0 (Fixed Irrigation—FX), 7, 14, and 21 days were applied. Nine treatments were studied in BA and five in MG. The water available in the soil was reduced to 44% for frequencies of shifting plant-row side irrigation of 7 days, 50% for 14 days, and 85% for 21 days, compared to the soil water availability at field capacity. Partial water deficit in the soil through the PRD technique did not significantly reduce the total root length, effective root depth, and root effective horizontal distance of the papaya Tainung genotype. However, PRD treatments showed leaf abscission, which resulted in reduced leaf area and NDVI values, especially in the MG experiment. Papaya yield and fruit quality were not affected. However, except for PRD 21 35%, irrigation water depth reduced to 50 and 35% under PRD increased crop water productivity (CWP) in papaya plants. Thus, the PRD technique may save 35% of WID using the alternation of lateral shift irrigation of crop row every 7 days under water scarcity in semi-arid regions. The NDVI index was important to compare the papaya canopy vigor between the experimental areas studied. We also confirmed the potential of NDVI to monitor the vigor of papaya canopy, since we could notice the sensibility of NDVI to identify water stress in papaya in higher vapor pressure deficit (VPD) conditions occurred in October 2016 and January 2017 in Bom Jesus da Lapa-BA. Therefore, the PRD strategy can be a useful tool to save water in papaya cultivation under semi-arid conditions.

## Introduction

Developing countries are expected to be more negatively affected by ongoing climate changes, mainly owing to rising temperatures and extreme drought episodes^1,2^. Brazil, a developing country, is among the ten countries with the largest irrigation area in the world^3,4^. From 1960 to 2015, irrigated areas in Brazil increased substantially, from 462 thousand hectares to 6.95 million hectares (Mha), and can expand more than 45% until 2030, reaching 10 Mha^3^. However, in both public and private irrigation projects, the consumption of water without technical criteria by farmers has negatively contributed to water resources sustainability^5,6^. Thus, these scenarios point to the urgent search for strategies of rational use of water in irrigated agriculture, especially considering that irrigation uses about 70% of all the freshwater consumed in the world^7^. The partial root-zone drying (PRD) technique has been showing as an alternative to increase crop water productivity (crop yields divided by the amounts of water supplied^8^) when compared to the conventional methods of irrigation management^6,9–13^. PRD consists of alternating the root system's irrigated side, i.e., while one part of the roots is irrigated, another part is exposed to soil drying^9^. According to previous^14^, drying the soil on one side induces the roots to produce abscisic acid, which is translocated through the xylem vessels to the leaves, reducing stomatal opening and transpiration.

Under a semi-arid condition in northern Iran, orange trees subjected to the PRD technique using 50% and 75% of full irrigation, alternating the side in each irrigation event, did not show reductions in fruit yield and quality^15^. Additionally, orange orchard subjected to irrigation depths of 50% and 100% of crop evapotranspiration (ETc), with the lowest irrigation depth applied in the PRD regime, increased fruit yield by 20% and 10% in 2013 and 2014, respectively^16^. Moreover, research carried out with banana cultivation, regardless of the soil type, showed that the interval of alternation of the irrigated side was decisive in reducing the volume of water applied, so that frequencies of 14 and 21 days caused substantial reductions in banana yield compared to 7 days^6^.

Although several studies have been conducted on the PRD technique, there are still knowledge gaps for different types of soil, climate and crops, to determine the feasibility of the PRD technique to farmers^17,18^, especially for some fruit trees, such as papaya^10,13,19^. Few studies have considered PRD techniques in papaya plants under controlled conditions (i.e., under greenhouse condition^9,10^), while PRD studies under papaya field conditions have just recently started^10,13,19,20^. This study reported that the PRD technique using irrigated side alternation frequency each 7–14 days with a 35% reduction in the irrigation depth could be a feasible technique to increase water use efficiency under semi-arid conditions.

The lack of high-throughput phenotyping tools is one of the bottlenecks that directly limit physiological measurements under field conditions^21^. However, spatial analysis techniques such as remote sensing analysis can provide useful information on a spatial manner's physiological performance. Remote sensing applications, mainly using vegetation indexes, such as the Normalized Difference Vegetation Index (NDVI), have shown potential in identifying and monitoring vegetation condition. This vegetation index is widely used to monitor crops, indicate production's potential, and guide crop management decisions^22^. The NDVI also improved methods for actual evapotranspiration prediction^23^, showing its potential to investigate the PRD technique's effects.

In 2017, the Brazilian production of papaya reached 1,057,101 Megagram (Mg), and the Northeast region was the largest producer (628,404 Mg), followed by the Southeast region (368,412 Mg). In the Northeast region, Bahia occupies the first position in production (368,875 Mg), and, of this total, 33.00% are produced in municipalities of the semi-arid region. The state of Minas Gerais occupies the fifth position in production in Brazil, with 71.37% coming from the semi-arid region^24^.

Given the substantial production of papaya in the semi-arid region, precise and technical information on irrigation management strategies is relevant. It will maximize water use efficiency in irrigated fruit production, focusing on alternative strategies for more sustainable production. In this context, this work is a step forward to a previous study^13^, aiming at improving our knowledge regarding PRD strategy in plant papaya under field conditions. This study aimed to evaluate the effects of the PRD technique on soil water regimes for different frequencies of shifting plant-row side irrigation as well as on the plant traits and fruit quality (specifically on titratable acidity—TA, total soluble solids—TSS, and TSS/TA ratio) of papaya cultivated in the semi-arid region of Bahia and the Minas Gerais States of Brazil. Also, we use conventional analysis to complement and better understand the impacts of PRD on papaya culture, and we also investigate it through a spatialized analysis using images from the Sentinel 2 and PlanetScope satellites. Thus, we hypothesized that decreasing the frequency of shifting plant-row side irrigation decrease the water availability, thereby increase root growth traits at the expense to reducing the shoot growth, fruit yield and quality and water productivity.

## Material and methods

### Experiments areas

**Figure 1:**
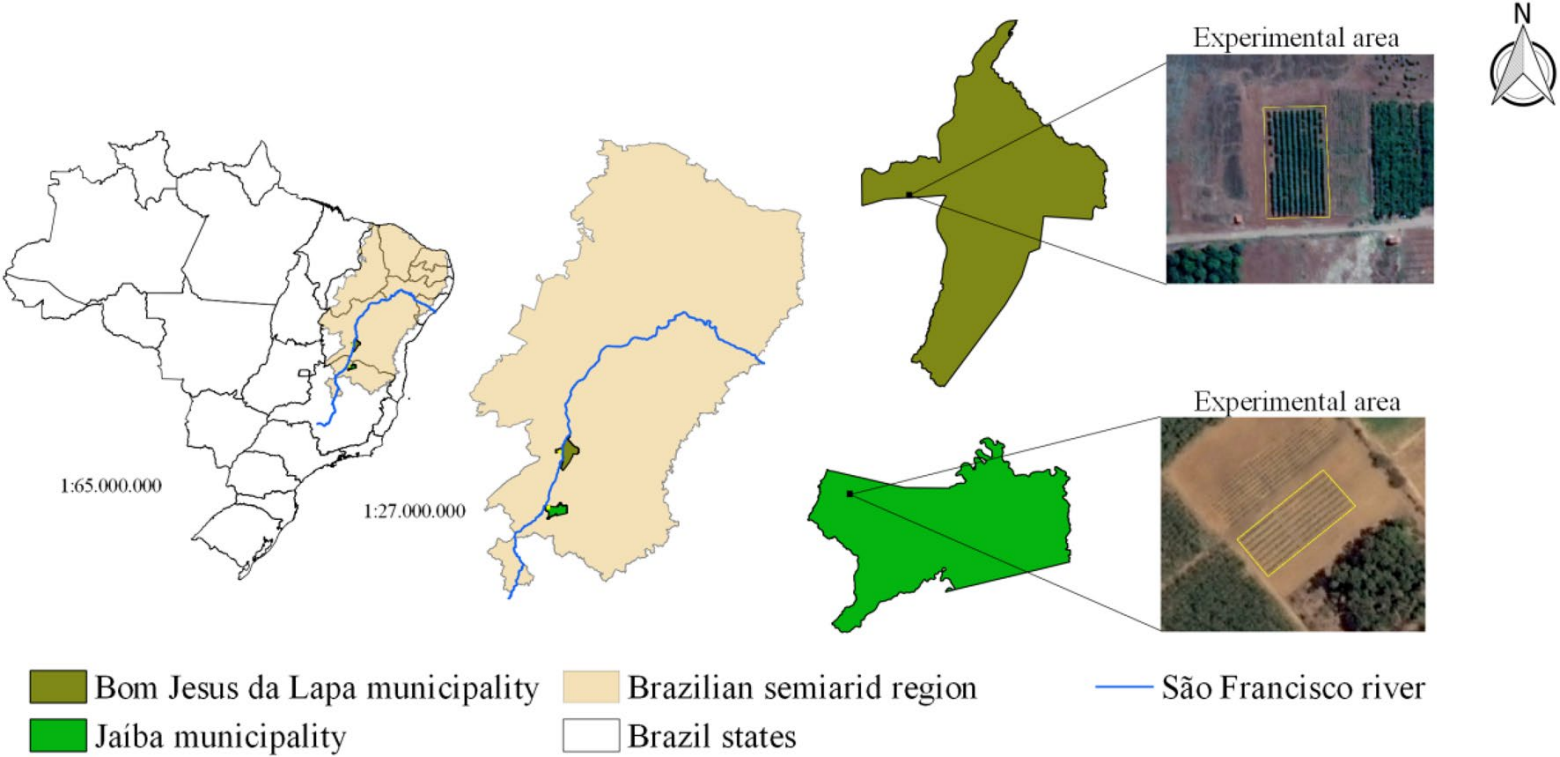
Location of the experiments showing the cities which each one is inserted, the proximity of both in relation to the São Franscisco river and their location in relation to the Brazilian semiarid region. The figure was elabored using the open source software QGIS^25^ using the Basemap: Google Satellites (obtained through QuickMapServices QGIS plugin), Map data ©2015 Google (https://www.google.at/permissions/geoguidelines/attr-guide.html).

**Figure 2:**
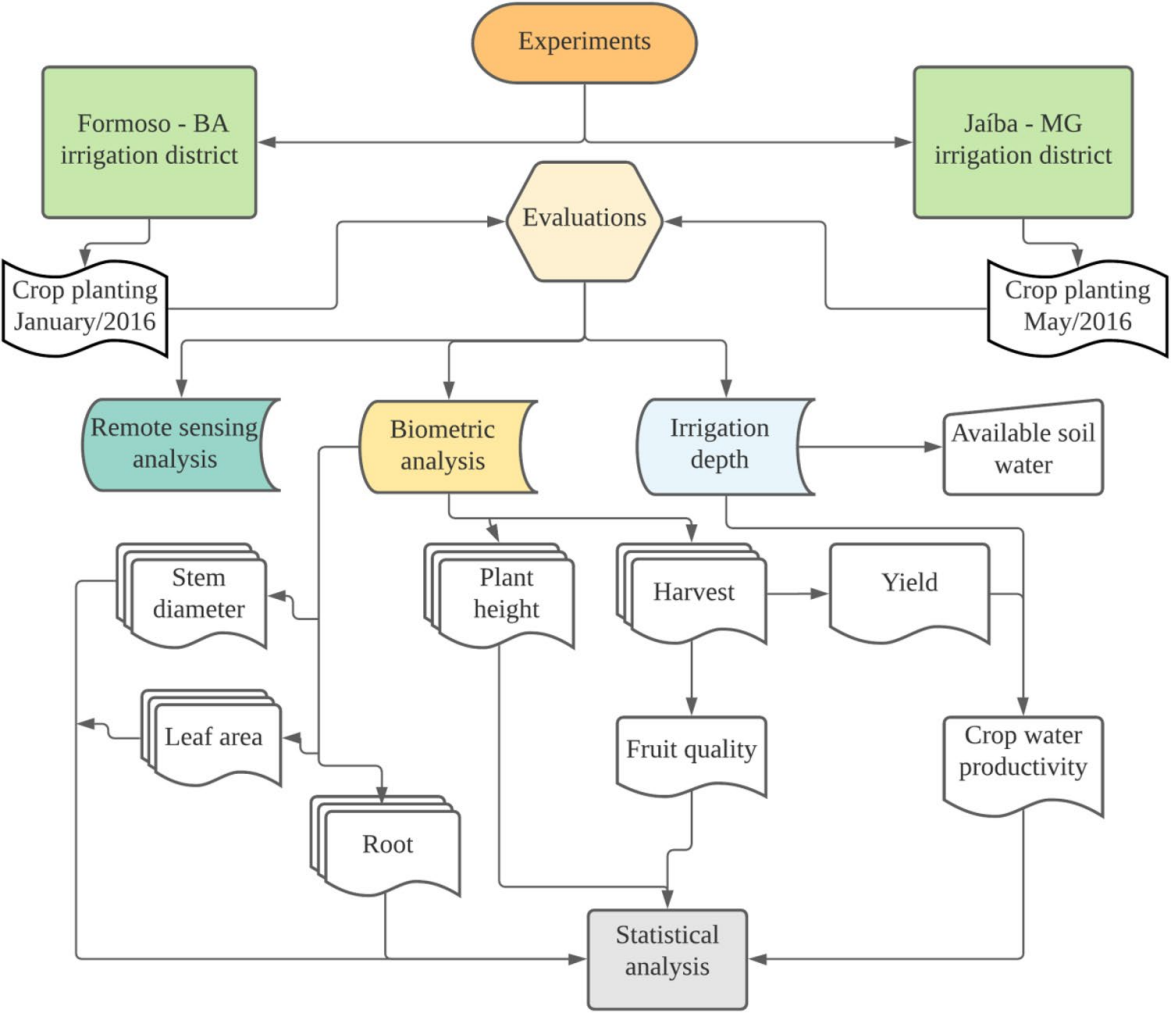
Step-by-step flowchart to show the stages of implementation, collection and analysis of data from the experiments.

The experiments were carried out in the Formoso Irrigation District (Bom Jesus da Lapa city) state of Bahia (BA) and Jaíba Irrigation District (Jaíba city) state of Minas Gerais (MG). Both sites are in two important regions of papaya production in Brazil (Fig. 1). The main methodological steps of collecting and processing data related to the experiments are presented in Fig. 2.

### Experiment located in Bahia state

The experiment was carried out from January 2016 to May 2017 [experimental field/Federal Institute of Education, Science and Technology (IF Baiano), 13°15′29″ S and 43°33′7″ W, and altitude of 441 m] (Fig. 1). According to Köppen’s classification, the climate is BSh (hot climate of Caatinga), with rainy summer and dry winter^26^.

The soil of the experimental area is classified as *Latossolo Vermelho-Amarelo* (Oxisol) with sandy clay loam textural class^27^. The soil moisture content in field capacity was 0.290 cm^3^ cm^−3^, (ψ_soil_ = −10 kPa), and the moisture content in permanent wilting point was 0.185 cm^3^ cm^−3^, (ψ_soil_ = −1500 kPa). The field capacity and permanent wilting point were determined according to the methodology proposed by previous study^28^, from 5 samples at depth 0.0–0.4 m from the experimental area.

**Figure 3:**
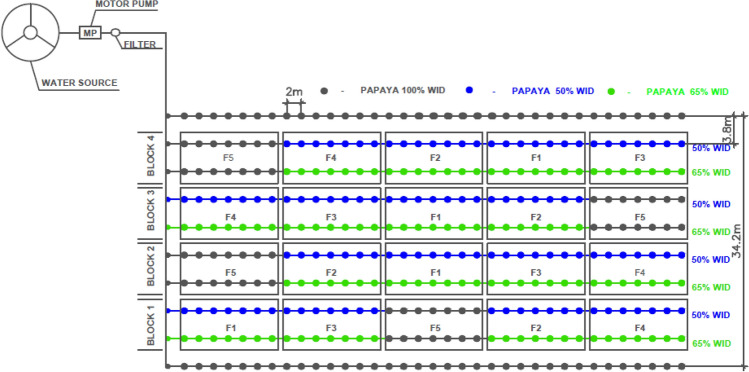
Treatments in the field conditions: F1—plant row side-shifting-frequency of 7 days, F2—plant row side-shifting-frequency of 14 days, F3—plant row side-shifting-frequency of 21 days, F4—only one plant-row side irrigated and F5—Both plant-row sides irrigated (100% WID).

*Carica papaya* L. seedlings (Tainung 01 genotype, from ‘Formosa’ group) were transplanted on January 5, 2016, at a spacing of 3.8 m × 2.0 m. The experiment followed a randomized block design with nine treatments and four replicates. Each replicate consisted of seven plants, with five usable plants. The treatments were based on the reduction of 50 and 35% of the full water irrigation depth (WID) and frequencies of shifting plant-row side irrigation of 0 (Fixed Irrigation—FX), 7, 14, and 21 days. Zero frequency represents water application on only one side of the plant [fixed irrigation (FX)] during the crop cycle (Fig. 3). As a result, it was possible to obtain the following combinations: PRD 7 50%—alternation every 7 days along the whole cycle, with 50% reduction in WID; PRD 7 35%—alternation every 7 days along the whole cycle, with 35% reduction in WID; PRD 14 50%—alternation every 14 days along the whole cycle, with 50% reduction in WID; PRD 14 35%—alternation every 14 days along the whole cycle, with 35% reduction in WID; PRD 21 50%—alternation every 21 days along the whole cycle, with 50% reduction in WID; PRD 21 35%—alternation every 21 days along the whole cycle, with 35% reduction in WID; FX 50%—fixed irrigation on one side of the plant with 50% reduction in WID; FX 35%—fixed irrigation on one side of the plant with 35% reduction in WID; and FU Control—full irrigation, i.e., application of 100% WID throughout the cycle.

### The experiment located in Minas Gerais state

**Figure 4:**
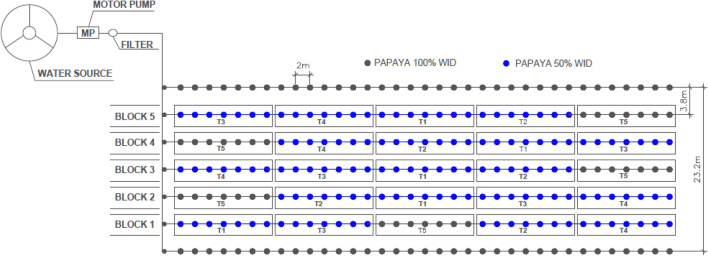
Placement of treatments in the field: T1—plant row side-shifting-frequency of 7 days (50% WID), T2–plant row side-shifting-frequency of 14 days (50% WID), T3—plant row side-shifting-frequency of 21 days (50% WID), T4—only one plant-row side irrigated (50% WID) and T5—Both plant-row sides irrigated (100% WID).

In Minas Gerais (MG), the experiment was carried out from May 2016 to September 2017. It was conducted in the experimental field of the Agricultural Research Company of Minas Gerais (EPAMIG), in Jaíba city—MG, located in the semi-arid region of Northern Minas Gerais, (15°07′47″ S, 43°57′0.5″ W, and altitude of 551 m; Fig. 4). The climate of the experimental site is classified as BSh, according to the Köppen-Geiger classification^29^.

The soil of the experimental area is a sandy loam, Typic Hapludalf soil^27^. The soil moisture content in the field capacity was (ψ_soil_ = −10 kPa) of 0.205 cm^3^ cm^−3^ and moisture content in the permanent wilting point (ψ_soil_ = −1500 kPa) of 0.135 cm^3^ cm^−3^.The field capacity and permanent wilting point were determined according to the methodology proposed by previous study^28^, from 5 samples at depth 0.0–0.4 m from the experimental area. *Carica papaya* L. seedlings (Tainung 01 genotype, from ‘Formosa’ group) was transplanted on May 24, 2016, at a spacing of 3.8 m × 2.0 m.

The experiment followed a randomized block design with five treatments and five replications. Each replication had seven plants in each planting row, with five usable plants, and between replicates, one plant was considered a border. The alternating frequencies of plant-row side irrigation were 0 (Fixed Irrigation—FX), 7, 14, and 21 days, and the zero-frequency represented water application on only one side of the plant during the papaya cultivation cycle (Fig. 3). The treatments studied were: PRD 7 50%—alternation every 7 days along the whole cycle, with 50% reduction in WID; PRD 14 50%—alternation every 14 days along the whole cycle, with 50% reduction in WID; PRD 21 50%—alternation every 21 days along the whole cycle, with 50% reduction in WID; FX 50%—fixed irrigation on one side of the plant with 50% reduction in WID; and FU Control—full irrigation, i.e., application of 100% WID throughout the cycle.

### Irrigation

A drip irrigation system with two lateral lines for row crop was adopted for all treatments, except for the FX, for which was adopted one lateral line. A total of six pressure-compensating emitters, with a flow rate of 4 L h^−1^, were installed in each plant, three on each side. The lateral line was positioned at a 0.25 m distance from the planting row. This irrigation system configuration allowed an average wetted area of 38% and a distribution uniformity of 95%.

Irrigation was applied daily to raise the soil water content to the superior limit of available water (field capacity). This soil water replenishment was based on the crop water evapotranspiration that was obtained according to the reference evapotranspiration by the modified Penman–Monteith method^30^ considering the crop coefficients recommended by previous study^31^ and the localization factor (Eq. 1). The values of crop coefficient were established according to the vegetative stage of the papaya. Crop coefficient varied linearly between 0.63 and 1.05 through the period of the experiment. Reference evapotranspiration was calculated daily using the data provided by the automatic meteorological stations from the National Institute of Meteorology (INMET, OMM: 86,672, and OMM: 86,720 codes), located in Bom Jesus da Lapa—BA and Jaíba city—MG, respectively.

1 $$ETc=ETo Kc Kl$$

where ETc, crop evapotranspiration for localized irrigation, mm d^−1^; ETo, reference evapotranspiration, mm d^−1^; Kc, crop coefficient, dimensionless; Kl, location factor, dimensionless.The location factor was calculated according to Eq. (2)^2^.

2 $$Kl=0.10 (SAP{)}^{0.5}$$

where Kl, location factor, dimensionless; SAP, shaded area percentage, %.

The time for irrigation for FU and all PRD 50% treatments in the experiment carried on Minas Gerais State has obtained by the ratio between ETc (mm) from Eq. (1) regarding the area (m^2^) of one plant and the total flow rate (L h^−1^) from six emitters per plant^33^. The irrigation time for PRD 35% treatments in the experiment carried in Bahia State was 30% greater than the time calculated in the same way as FU and PRD 50% in the experiment carried in Minas Gerais. The two lateral lines remained on during the calculated irrigation time for FU treatment. Only one lateral line remained on during the irrigation events, in all other treatments either for PRD 50% or for PRD 35%. This operation was accomplished by using manual valves at the beginning of lateral lines.

Electric resistance sensors (Watermark. Irrometer Company, USA) were installed at a depth of 0.25 m, at a radial distance of 0.10 m from the emitter, and at 0.25 m from the plant stem at both sides of it. The use of sensors evaluated soil water potential at two sides of the crop row in each treatment.

At all treatments, the soil water content was also evaluated as soil water availability by using Eq. (3).

3 $$SWA=\left[\frac{{\theta }_{\text{at}}\text{ - }{\theta }_{\text{pm}}}{{\theta }_{\text{cc}}\text{ - }{\theta }_{\text{pm}}}\right]{100}$$

where SWA, soil water available, %; θ_at_ actual soil water content, cm^3^ cm^−3^; θ_cc_, soil water content at field capacity, cm^3^ cm^−3^; θ_pm_, soil water content at the wilting point, cm^3^ cm^−3^.

### Cultivation practices

The seedlings were produced in a protected environment at Embrapa Cassava and Fruits, Cruz das Almas, BA, Brazil and subsequently taken to the experimental area. Three seedlings were transplanted per hole. The plants were arranged in 10 single rows, each row with 35 plants. Sixty days after the transplanting date, thinning was performed, and the most vigorous hermaphrodite plant was selected per hole. The use of plants in the present study complies with international, national and/or institutional guidelines.

Weeds were controlled by manually weeding in the plant rows and mechanically weeding between rows using a tractor-mounted mower.

As soon as the level of economic losses was reached, pests and diseases were controlled through the chemical method, by spraying insecticides of the Avermectin chemical group and Kumulus, and fungicides of the Score chemical group according to the recommendations of the manufacturers.

The fertilization of papaya was performed by fertigation events distributed at 15-days intervals during the months of the experimental treatments based on soil chemical analysis and recommendations from specific literature^34^.

### Papaya growth traits

In both experiments, 5 papaya plants per block were evaluated for height, stem diameter, and leaf area. Plant height was obtained by measuring the length from the soil surface to the apex of the plant using a graduated ruler. Stem diameter was determined from the circumference measured at 0.10 m height from the soil surface using a graduated ruler. Leaf area of the canopy was determined from the length of the central lobes of all leaves of the plant, according to Eq. (4), proposed by previous study^35^.

4 $$LA = 0.0947L^{2.7352}$$

where LA, single leaf area, m^2^; L, length of the central lobe, m.

Root samples were collected at 240 days after transplanting the papaya seedlings to the experimental area, by the monolith method^36^, longitudinally to the drip line. A sample of root of 1,000 cm^3^ were collected each block at the combinations of distances of 0, 0.20, 0.40 and 0.60 m from the plant stem and depths of 0.0–0.15, 0.15–0.30, 0.30–0.45 and 0.45–0.60 m. After collection, the samples were placed in plastic bags and sent to the Irrigation and Fertigation Laboratory from Embrapa Cassava and Fruits. The roots from each sample were separated from the soil by washing with water and exposed to natural drying on a workbench. After drying, the roots were scanned using a transparent plastic, and the images were saved as TIFF (Tagged Image File Format) files. The TIFF files were subjected to the Rootedge software^37^ to determine the total cumulative root length, effective depth, and an effective horizontal distance of the root system.

### Fruit yield and quality

In both experiments, fruits were harvested weekly when they had less than 15% of the yellow peel color surface (maturity stage I)^38^. At harvest, they were weighed and identified in their respective treatments. The values obtained in the weighing procedures were converted to yield. The crop water productivity (CWP) was assumed as the ratio between fruit yield and crop evapotranspiration^39^.

Four months after the beginning of the papaya harvest, four fruits were randomly selected in each treatment in both experiments. These fruits were taken to the Plant Physiology and Postharvest Laboratory from Embrapa Cassava and Tropical Fruits. It was evaluated the chemical attributes: titratable acidity (TA) and soluble solids (SS). The fruits were sanitized in the laboratory by washing with neutral detergent and subsequently stored until reaching maturity stage 5 (more than 75% of yellow peel).

Titratable acidity (TA) was determined by weighing 1 g of the fruit pulp in a beaker and then adding 30 mL of distilled water and three drops of phenolphthalein. Subsequently, the mixture was titrated with 0.1 N sodium hydroxide (NaOH) solution until it reached a light pinkish color, using the Metrohm-775 Dosimat semi-automatic digital burette. The analyses were performed in duplicate, expressing the results in g of citric acid/100 g of pulp^40^. Soluble solids were quantified by the direct refractometric reading of the degrees Brix of the sample, using a Kitler Mod. 113 handheld refractometer (0–32°), with a range of 0.2°. After, the SS/TA ratio was calculated.

### Statistical analysis

The data of crop growth, titratable acidity (TA), total soluble solids (SS), SS/AT ratio of fruits, fruit yield, and water productivity of papaya from experiments carried in BA and MG were evaluated statistically for each experiment by analysis of variance where the null hypothesis was verified the by F test. When it was significant, the means of treatments were submitted to the cluster analysis by the Scott-Knott test at a 5% probability level.

### Remote sensing analysis (RS)

Two study approaches were performed using images from orbital platforms in the experiments of the present study. The first analysis was a temporal approach encompassing the entire experimental area (i), which was realized for both experimental areas, and the second analysis was a temporal and spatial approach to verify the crop conditions submitted to the different techniques of PRD (ii). This analysis was more spatially detailed. Therefore, it was performed only for the experiment located in Bom Jesus da Lapa—BA, due to the larger size of the sample area. Both analyses calculated the Normalized Difference Vegetation Index (NDVI) to observe the vegetation's condition in the experiments.

#### Remote sensing analysis (i)

This analysis aimed to understand how the leaf cover was changed and its relationship with the meteorological variables throughout the papaya cycle. For the first analysis, the constellation Sentinel 2 (Sentinel 2A and Sentinel 2B) was used since the purpose was to evaluate the condition of the vegetation considering the entire area over the entire period of the two experiments, allowing a comparison of the vegetative vigor between experiments. This constellation of satellites was used since it has a high frequency of images (temporal resolution of 10 days and frequency of 5 days with both satellites)^41^ with sufficient spatial resolution to acquire information regarding the areas samples.

The Sentinel 2 constellation satellites have the MultiSpectral Imager (MSI) sensor capturing the electromagnetic radiation reflected from the surface in 13 spectral bands^42^. For the present study, only the bands referring to near-infrared—NIR and red (RED) were used, both with 10 m of spatial resolution. The band referring to the NIR (Band 8) captures radiation referring to the amplitude of the wavelength from 780 to 900 nm and the band of the red (Band 4) from 650 to 680 nm^43^. We acquired seven free cloud images referring to the LPF tile for Bom Jesus da Lapa/BA experiment and seven free cloud images referring to the LPD tile for the experiment located in the municipality of Jaíba/MG.

The NIR and RED bands were used to calculate the NDVI proposed by previous study^44^, according to Eq. (5).

5 $$\text{NDVI }\frac{\left({\rho}_{\text{NIR}} \,- \, {\rho}_{\text{RED}}\right)}{\left({\rho}_{\text{NIR}} \,+ \, {\rho}_{\text{RED}}\right)}$$

where ${\rho}_{\text{NIR}}$ and ${\rho}_{\text{RED}}$ refer to the reflectance of the infrared and red spectral bands.

After calculating the NDVI, we calculated the average value of this index for each image date. We did this to understand the temporal variability of the papaya NDVI with the weather conditions of the day of the satellite passage and to be able to compare the average NDVI values between the experiments.

#### Remote sensing analysis (ii)

For analysis ii (remote sensing analysis ii), since a greater spatial resolution was necessary, the PlanetScope satellite constellation was used. This constellation is composed of more than 150 nanosatellites (CubeSats)^45^, most of which have a synchronous orbit with the sun^46^.

The sensor present in these CubeSats captures electromagnetic radiation in four spectral bands, which are: blue (455–515 nm), green (500–590 nm), red (590–670 nm), and near-infrared (NIR, 780–860 nm), with a spatial resolution of 3 m. For this study, only the bands referring to NIR and RED were used since the purpose was to calculate the NDVI of papaya with spatial details to infer the vegetation's condition under the different PRD techniques used in the experiment of Bom Jesus da Lapa/Bahia, Brazil.

We downloaded nine cloud-free images referring to the PlanetScope Ortho Scene Product. This product has a processing level denominated as Level-3B, which already has an atmospheric correction and, therefore, is already in surface reflectance^45,47^. The atmospheric correction method used for this product is the 6SV2.1 radiative transfer code.

## Results

### Soil water dynamic

During the experiment, the total cumulative precipitation was 767 mm in Bom Jesus da Lapa—BA and 719 mm in Jaíba—MG (Table 1). The highest monthly precipitation values were recorded in January 2016 (350 mm) and November 2016 (286 mm), in Bom Jesus da Lapa—BA and Jaíba—MG, respectively. The monthly vapor pressure deficit (VPD) ranged from 1.9 up to 3.4 kPa in the BA experiment and from 1.4 up to 2.9 kPa in the MG experiment.

**Table 1:** Monthly mean values of vapor pressure deficit (VPD in kPa) and monthly cumulative values of water irrigation depth (WID in mm) and precipitation (P in mm) along the months of experiment in Bom Jesus da Lapa-BA and Jaíba-MG.

Month/Year	Bom Jesus da Lapa-BA					Month/Year	Jaíba-MG		
	VPD (kPa)		WID		*P*		VPD (kPa)	WID	*P*
			(mm)					(mm)	
Jan/16	1.9		0		350	May/16	1.5	29	0
Feb/16	2.0		27		0	Jun/16	1.4	30	0
Mar/16	2.2		54		0	Jul/16	1.4	36	0
Apr/16	2.6		51		0	Aug/16	1.7	40	0
May/16	2.4		86		0	Sep/16	2.1	43	0
Jun/16	2.6		89		2	Oct/16	2.1	69	28
Jul/16	2.5		92		0	Nov/16	1.5	71	286
Aug/16	3.2		137		0	Dec/16	1.6	103	86
Sep/16	3.0		157		0	Jan/17	2.3	136	106
Oct/16	3.2		169		0	Feb/17	1.5	90	116
Nov/16	2.6		43		101	Mar/17	1.9	166	82
Dec/16	2.4		91		63	Apr/17	2.1	141	1
Jan/17	3.4		102		17	May/17	2.0	123	14
Feb/17	2.6		137		52	Jun/17	2.1	122	0
Mar/17	2.7		48		132	Jul/17	1.9	144	0
Apr/17	2.4		84		46	Aug/17	2.6	134	0
May/17	2.9		78		4	Sep/17	2.7	110	0
Total			1446		767	Total		1587	719

**Figure 5:**
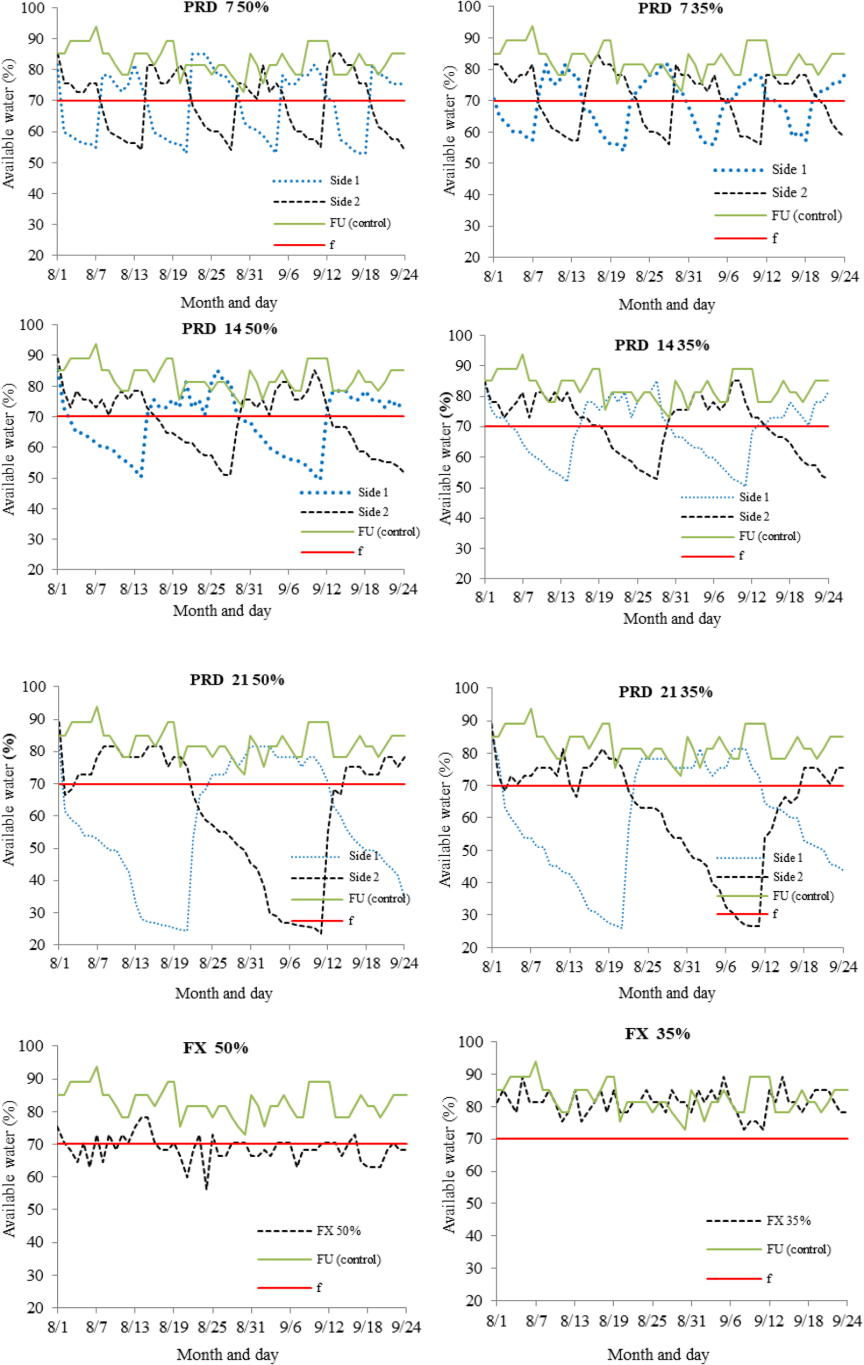
Available water in the soil under the different PRD irrigation regimes: partial rootzone drying, with 50 and 35% reductions in the water irrigation depth (WID), alternating at 7, 14 and 21 days; FX: fixed irrigation on one side of the rootzone, with 50 and 35% reductions in WID; FU: full irrigation, 100% of WID, and f: lower limit of soil water availability for papaya, in Bom Jesus da Lapa–BA.

**Figure 6:**
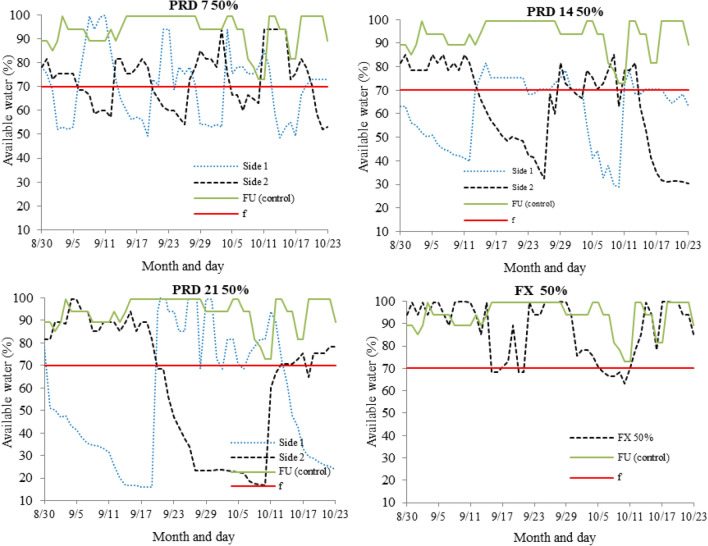
Available water in the soil under the different PRD irrigation regimes: partial rootzone drying, with 50% reduction in the water irrigation depth (WID), alternating at 7, 14 and 21 days; FX: fixed irrigation on one side of the rootzone, with 50% reduction in WID; FU: full irrigation, 100% of WID, and f: lower limit of soil water availability for papaya, in Jaíba–MG.

The PRD 4 50% provided 55% of soil water available in the BA experiment, and 50% in the MG experiment on the side of crop row subjected to drying. These values correspond to reductions of 15% and 20% below the critical soil water available (f = 70%), corresponding to the critical moisture or potential assumed for papaya (Figs. 5 and 6). In the PRD 7 35% treatment in Bom Jesus da Lapa, the average value of soil water available below the critical one (70%) was approximately 43.5% (Fig. 5).

Due to the longer time subjected to drying, the highest values of reduction of SWA in comparison to the limit required by papaya (70%), in both experiments, occurred in the PRD 21 treatment with 50 and 35% reductions in WID, followed by the PRD 14 treatment with 50 and 35% reductions (Figs. 5 and 6). In the BA experiment, in the treatments with irrigated side alternating in periods of 21 and 14 days, the values of soil water content on the side of the plant subjected to drying reached means correspondent to SWA of 25 and 50%, respectively. For these same alternating periods of irrigated side in the MG experiment, the values of soil water available reached means close to 15% and 30%, respectively (Fig. 5).

In both experiments, the soil water available in the FU Control treatment remained with values higher than the critical limit of soil water available (Figs. 5 and 6), as required by papaya. In the FX treatments with 50 and 35% reductions in WID (BA experiment), the values of soil water available on the permanently irrigated side remained between 57 and 80%. In contrast, in the MG experiment, the soil water available ranged between 60 and 100% in the FX 50% treatment.

### Papaya growth

Plant height was the most influenced growth variable by the treatments, differing among treatments at 80 and 240 days after transplanting (DAT) in the BA experiment and only at 90 DAT in the MG experiment. In the BA experiment, at 80 DAT, the highest plant height was found in the PRD 14 50% treatment, differing from the others (Table 2). At 240 DAT, the highest plant height was observed in the treatments FU, PRD 7 with 50 and 35% reductions in WID, followed by the treatments PRD 21 with 50 and 35% reductions in WID, and differing from the treatments PRD 14 and FX with 50 and 35% reductions in WID, which reached the lowest plant height (Table 2).

**Table 2:** Mean of plant height, stem diameter and leaf area of papaya cultivated under PRD: with alternation at 7, 14 and 21 days; FX: fixed irrigation on one side of the rootzone; and FU: full irrigation in different periods (days after transplanting, DAT), in Bom Jesus da Lapa-BA and Jaíba-MG.

Treatments	Bom Jesus da Lapa-BA			Jaíba-MG		
	80 DAT	140 DAT	240 DAT	90 DAT	145 DAT	200 DAT
**Plant height (m)**						
PRD 7 35%	1.44b	2.33a	2.86a			
PRD 14 35%	1.32b	2.19a	2.67b			
PRD 21 35%	1.48b	2.29a	2.84a			
FX 35%	1.44b	2.23a	2.65b			
PRD 7 50%	1.54b	2.28a	2.91a	0.86a	1.13a	1.37a
PRD 14 50%	1.72a	2.28a	2.65b	0.90a	1.09a	1.34a
PRD 21 50%	1.25b	2.25a	2.81a	0.82b	1.10a	1.32a
FX 50%	1.48b	2.21a	2.64b	0.86a	1.10a	1.32a
FU	1.54b	2.50a	2.94a	0.96a	1.22a	1.51a
*p* (F test)	0.00	0.30	0.04	0.00	0.26	0.07
CV (%)	8.51	8.67	7.68	6.64	8.72	7.92
**Stem diameter (m)**						
PRD 7 35%	0.195b	0.345a	0.395a			
PRD 14 35%	0.185b	0.335a	0.390a			
PRD 21 35%	0.205a	0.342a	0.400a			
FX 35%	0.180b	0.352a	0.412a			
PRD 7 50%	0.212a	0.335a	0.422a	0.141a	0.200a	0.278a
PRD 14 50%	0.242a	0.332a	0.402a	0.147a	0.194a	0.278a
PRD 21 50%	0.157b	0.320a	0.410a	0.143a	0.194a	0.293a
FX 50%	0.190b	0.337a	0.412a	0.156a	0.231a	0.289a
FU	0.167b	0.387a	0.450a	0.152a	0.210a	0.288a
*p* (F test)	0.01	0.18	0.25	0.30	0.46	0.94
CV (%)	7.26	7.39	9.26	8.20	17.10	12.05
**Leaf area (m**^**2**^**)**						
PRD 7 35%	1.54a	5.93a	4.64a			
PRD 14 35%	1.07a	4.45b	3.62a			
PRD 21 35%	1.83a	5.03b	4.34a			
FX 35%	1.01a	5.90a	3.26a			
PRD 7 50%	1.80a	5.46a	4.79a	1.84a	2.38a	6.88a
PRD 14 50%	1.29a	4.29b	3.38a	1.93a	2.28a	6.86a
PRD 21 50%	1.12a	4.46b	4.33a	1.74a	2.20a	6.24a
FX 50%	0.92a	4.78b	4.35a	2.22a	2.21a	6.43a
FU	1.91a	6.11a	5.21a	2.29a	2.97a	8.53a
*p* (F test)	0.12	0.04	0.15	0.17	0.61	0.34
CV (%)	23.15	43.22	25.31	19.70	34.33	22.76

Means followed by the same letter in the column did not differ statistically by Scott-Knott test at 5% probability level.

In the MG experiment, the PRD 21 50% treatment was the only one that differed from the FU treatment for plant height, with a reduction of 14.58%. This lower plant height can be justified by the 55% reduction in soil available water compared to the lower limit required by papaya (f = 70%) (Fig. 6).

At 80 DAT, the highest stem diameter was observed in the treatments, PRD 14 50%, PRD 7 50%, and PRD 21 35%, and the mean diameters in the other treatments did not differ, even considering the FU treatment (Table 2). There was no significant difference for stem diameters among treatments from 140 DAT in the BA experiment and 90 DAT in the MG experiment.

There was no significant difference between the treatments concerning papaya's leaf area, except at 140 DAT in the BA experiment. At 140 DAT, the largest leaf areas occurred in the treatments FU, PRD 7 (50 and 35%), and FX 35%, which differed from the others (Fig. 5). It is possible to observe a reduction of leaf area in all treatments in the BA experiment at 240 DAT (September 2016) compared to 140 DAT (May 2016).

### Remote sensing

**Figure 7:**
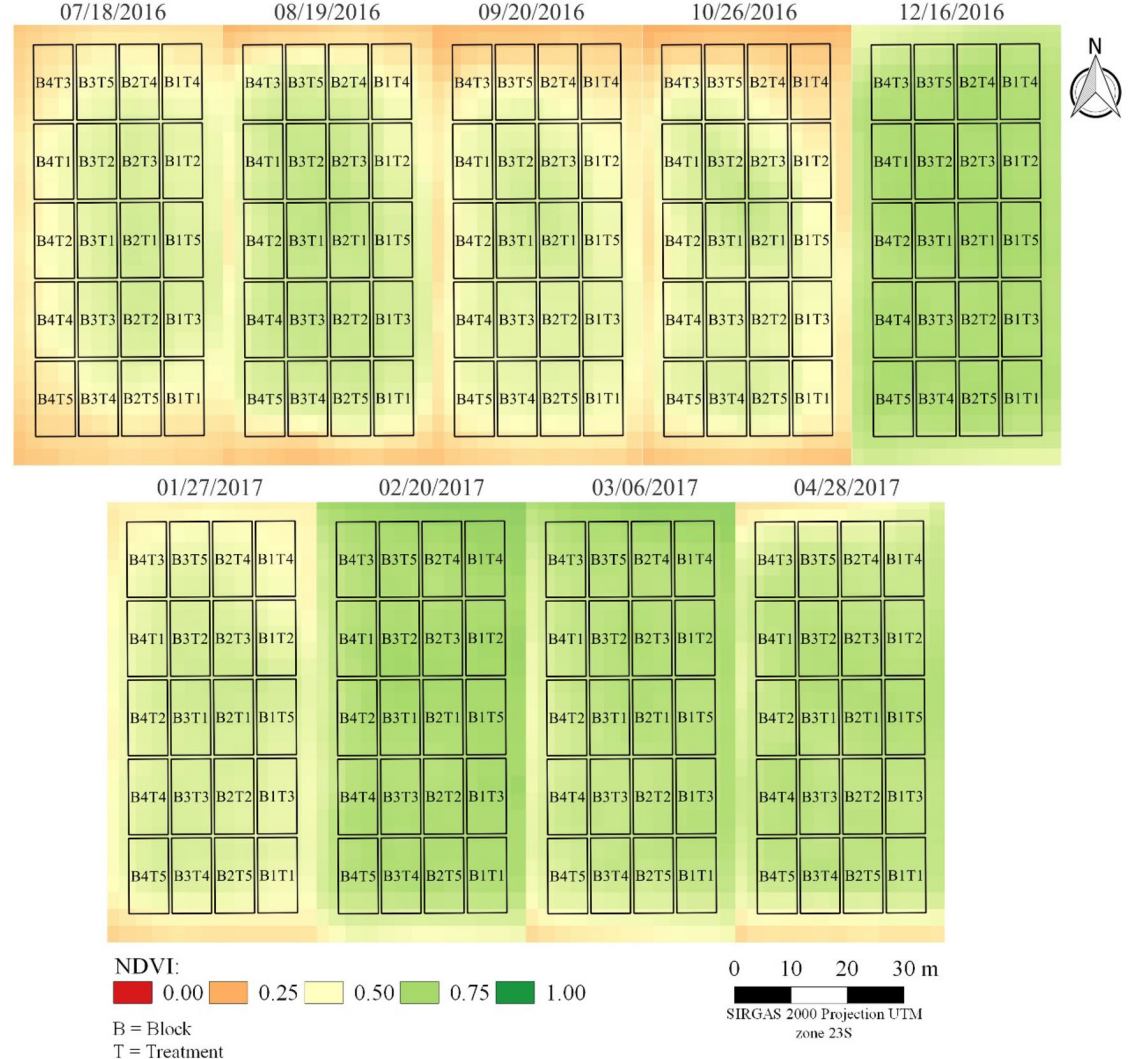
NDVI images from the PlanetScope constellation for the period of conduction of the experiment in Bom Jesus da Lapa, Bahia, Brazil.

**Figure 8:**
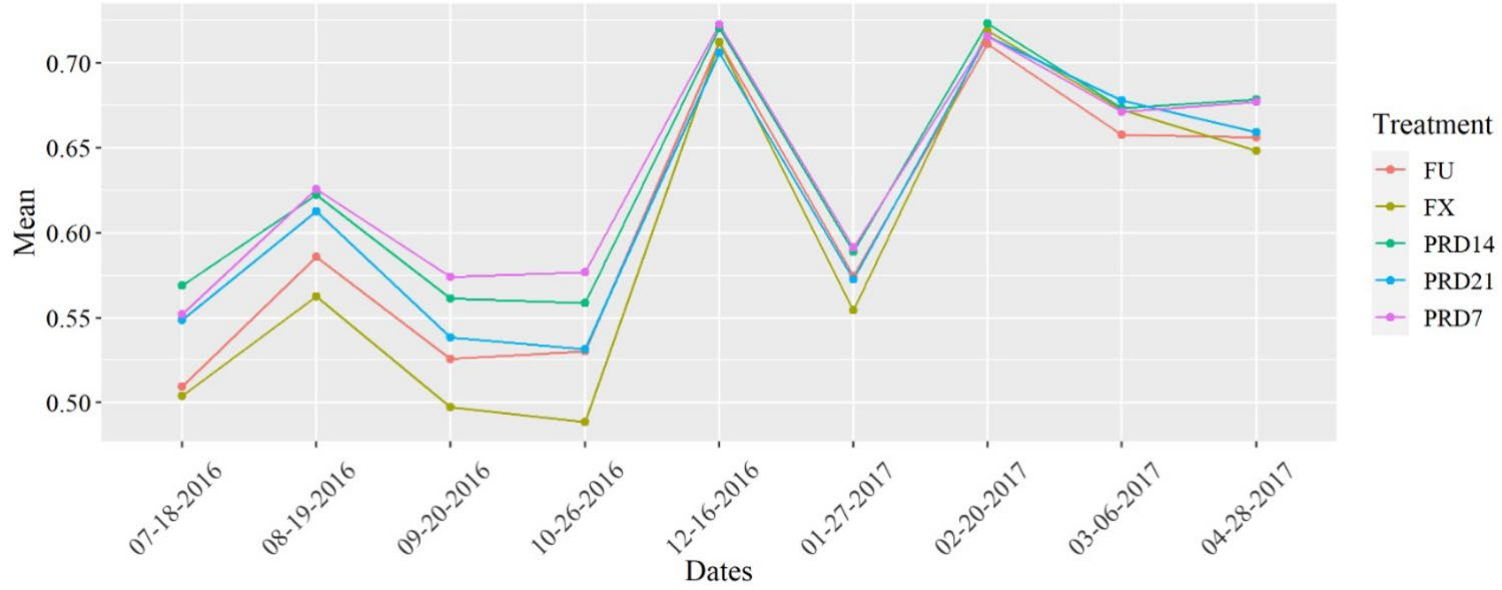
Temporal values of NDVI obtained from PlanetScope constellation in different PRD techniques, PRD 7—alternation every 7 days along the whole cycle; PRD 14—alternation every 14 days along the whole cycle; PRD 21—alternation every 21 days along the whole cycle; FX—fixed irrigation on one side of the plant; and FU Control-full irrigation.

Two scenarios were observed concerning the NDVI values of the experimental area of Bom Jesus da Lapa—BA (Fig. 7). The first scenario corresponds to the high temperature and vapor-pressure deficit (Table 1): greater thermal stress, images at 194 (07/18/2016), 227 (08/19/2016), 259 (09/20/2016), and 295 (10/26/2016) days after transplanting, respectively. The other scenario corresponds to the period of temperature and vapor-pressure deficit of milder air, images from 12/16/2016, 02/20/2017, 03/06/2017 e 04/28/2017, at 345, 387, 411, 425, and 478 days after transplanting, respectively, except for the image of January/2017, period on which there was an increase in vapor-pressure deficit (Table 1). The NDVI values from the two scenarios are directly associated with the papaya tree leaf area in different treatments (Fig. 8 and Table 2).

In the period of greatest thermal stress (from July/2016 to October/2016), the NDVI varied on average from 0.46 to 0.62, with the highest average values in the treatments with the frequency of alternation of the irrigated side of 7, 14, and 21 days, the lowest values occurred in the FX and FU treatments (Fig. 8).

**Figure 9:**
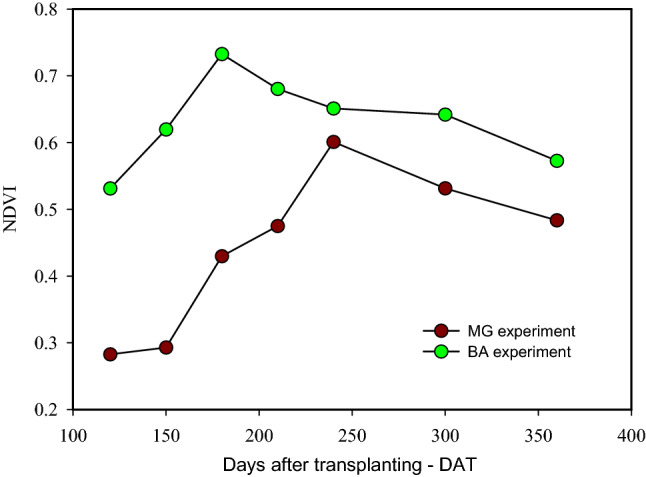
Mean NDVI values from Sentinel 2 for MG experiment and BA experiment.

NVDI observed in the experiment conducted in Bahia was greater than the one on the MG experiment, mainly from 120 to 240 DAT. This was the period of vegetative growth of the crop (Fig. 9).

### Papaya root system

There was no significant difference between the treatments studied for the root system's effective depth, effective horizontal distance of the root system, and total cumulative root length (Table 3). Thus, the use of the PRD technique did not cause losses in the growth of papaya roots. The root system's effective depth, that is, the depth corresponding to 80% of the total length of roots, varied between 0.42 m (PRD 21 50%) and 0.44 m (PRD 7 50%, PRD 14 50%, and FU).

**Table 3:** Effective depth, effective horizontal distance and total cumulative length of roots of papaya cultivated under PRD: with alternation at 7, 14 and 21 days and 50% reduction in WID; FX: fixed irrigation on one side of the rootzone, with 50% reduction in WID; and FU: full irrigation, 100% of WID, in Jaíba-MG.

Treatments	Effective depth	Effective horizontal distance	Total cumulative length
	(m)		
PRD 7 50%	0.44a	0.41a	6.359a
PRD 14 50%	0.44a	0.41a	6.806a
PRD 21 50%	0.42a	0.39a	6.632a
FX 50%	0.43a	0.43a	4.310a
FU	0.44a	0.41a	6.132a
*p* (F test)	0.82	0.13	0.11
CV (%)	5.73	5.38	21.73

Means followed by the same letter in the column do not differ statistically by Scott-Knott test at 5% probability level.

### Fruit yield, ratio SS/TA and crop water productivity

It is possible to verify a reduction of yield of treatments submitted to 50%WID in the MG experiment compared to the BA experiment. Such a decrease of yield is justified by the severe attack of mites (*Tetranychus urticae*) along with the vegetative and reproductive development of papaya plants.

The yields of the treatments subjected to the PRD technique in the BA experiment were higher than those in the MG experiment (Table 4). There was no significant difference in the mean fruit yield between the treatments FU, PRD 7 35%, and PRD 14 35%, which differed from those in the BA experiment. The lowest fruit yield occurred in the PRD 21 50% treatment (67,594 kg ha^−1^).

**Table 4:** Mean yield, mean weight, titratable acidity (TA), soluble solids (SS), SS/TA ratio of fruits and crop water productivity (CWP) of papaya cultivated under PRD: with alternation at 7, 14 and 21 days; FX: fixed irrigation on one side of the rootzone; and FU: full irrigation, in Bom Jesus da Lapa-BA and Jaíba-MG.

Treatments		Yield (kg ha^−1^)	Fruit weight (g)	TA (g citric acid/100 g pulp)		SS (%)		SS/TA	CWP (kg/m^3^)
**BA experiment**									
PRD 7 50%		70,564b	1,376.6a	0.12a		12.57a		99.78a	9.26a
PRD 7 35%		89,933a	1,167.4a	0.12a		12.27a		96.48a	9.08a
PRD 14 50%		70,253b	1,056.3a	0.10a		11.22b		107.20a	9.22a
PRD 14 35%		84,453a	1,136.3a	0.12a		11.67b		98.38a	8.52a
PRD 21 50%		67,594b	1,186.3a	0.09a		11.30b		116.10a	8.87a
PRD 21 35%		71,837b	1,144.0a	0.11a		11.90b		100.28a	7.25b
FX 50%		70,058b	1,129.5a	0.11a		11.75b		99.26a	9.19a
FX 35%		71,467b	1,374.7a	0.12a		12.45a		104.32a	7.21b
FU		96,218a	1,226.7a	0.12a		13.22a		110.52a	6.31b
*p* (F test)		0.00	0.06	0.18		0.03		0.25	0.02
CV (%)		14.43	18.59	13.09		6.60		10.70	15.93
**MG experiment**									
PRD 7 50%		60,464b	995.0b	0.33a		13.47a		41.27a	7.16a
PRD 14 50%		60,948b	972.0b	0.24b		13.90a		56.29a	7.22a
PRD 21 50%		54,200b	991.0b	0.30a		13.55a		45.39a	6.42a
FX 50%		59,977b	1,064.0ab	0.30a		12.67a		41.98a	7.10a
FU		78,542a	1,138.0a	0.25b		13.27a		53.99a	4.83b
*p* (F test)		0.00	0.00	0.04		0.37		0.06	0.02
CV (%)		15.18	5.31	13.55		6.27		16.18	15.93

Means followed by the same letter in the column do not differ statistically by Scott-Knott test at 5% probability level.

The treatments PRD 7, PRD 14, and FX, with a 50% reduction in WID, reached very similar yields, with values close to 70 Mg ha^−1^ in the BA experiment and 60 Mg ha^−1^ in the MG experiment. In all treatments, the lowest reduction in the applied WID and the shortest interval of alternation in the irrigated side promoted higher yield. In the treatments, PRD 7 35% and PRD 14 35%, the yields were 20 and 15% higher than those in the FX 35% treatment, respectively. This result demonstrates the superiority of plants subjected to the PRD technique compared to the irrigation in only one side of the plant (Table 4).

**Figure 10:**
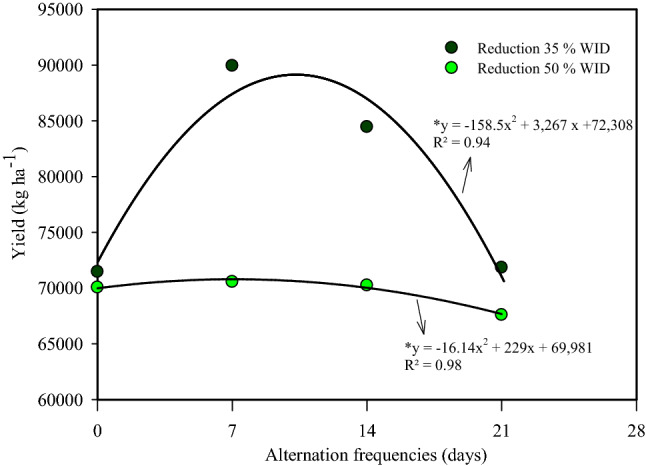
Relationship between the frequency of alternation on the irrigated side and productivity, in Bom Jesus da Lapa, Bahia, Brazil. *Significant by F test at 5% probability.

The treatments subjected to the PRD technique and FX with 50% reduction (BA and MG experiments) and a 35% reduction in WID (BA experiment) reached water saving levels of 723, 793.5, and 506 mm, respectively (Table 1). The interaction between the alternation frequency of the plant-row irrigated side and the percent of WID reduction showed a significant effect (p < 0.05) only for fruit yield (Table 5). Means of yields under alternating frequencies of 7 and 14 days with 50% WID differed from the ones with 35% WID of 21,53% and 16,81%, respectively (Table 5). A second-degree polynomial fitted the data of yield as a function of the frequency of alternation of the plant-row irrigated side (p < 0.05) for both 50 and 35% WID reduction (Fig. 10). The absence of alternation frequency (FX treatment) did not provide a significant increase (p < 0.05) of yield for the reduction of 35% WID when compared with the 50%WID reduction (Table 5, Fig. 10). However, there was an increase of yield for 35% WID reduction under 7 and 14 days of alternation frequency.

**Table 5:** Interaction between the frequency of alternation of the irrigated side versus the reduction of the WID for the yield variable, Bom Jesus da Lapa, Bahia, Brazil.

Reduction WID (%)	Alternation frequencies (days)			
	0	7	14	21
50	70,058a	70,564b	70,253b	67,594a
35	71,467a	89,933a	84,453a	71,837a

Means followed by the same letter in the column do not differ statistically by Tukey test at 5% probability level.

There was no statistical difference (p > 0.05) among treatments for titratable acidity (BA experiment) and total soluble solids (MG experiment). Treatments PRD 7 50%, PRD 7 35%, FX 35%, and FU resulted in larger means of total soluble solids (SS) in the BA experiment.

Treatments PRD 21 35%, IF 35% e FU carried in BA showed smaller means of crop water productivity (CWP) than the others. The larger differences of CWP among PRD and FU treatments concerned about PRD 7 50%, PRD 14 50%, and PRD 7 35%, with 2.94 kg/m^3^, 2.90 kg/m^3^, and 2.76 kg/m^3^, respectively (Table 4). The CWP was significantly larger for treatments submitted to a 50% reduction of irrigation water depths than CWP from FU treatment of the MG experiment.

## Discussion

Due to the absence of rainfall in eight months in the BA experiment and nine months in the MG experiment (Table 1), it was possible to characterize the PRD irrigation management based on the soil available water in the root-zone, monitored on both sides of the plant for the different treatments (Figs. 5 and 6). In addition to the occurrence of these rains in a few months, the intensity of these is generally high, which means that the crop is not able to take advantage of all the water that was precipitated in this short period of the year, which explains the irrigation depth of 71 mm (Fig. 3) in November 2016 in BA experiment. The soil moisture contents ranged between field capacity and permanent wilting point in the studied treatments during the experimental period.

### Effects of partial root-zone drying (PRD) technique on plant growth traits

Plant height was the most influenced growth variable by the treatments so that the highest plant height was found in the PRD 14 50% treatment at 80 DAT (Table 2) and in FU, PRD 7 with 50 and 35% reductions in WID at 240 DAT in the BA experiment. In the MG experiment, the PRD 21 50% treatment was the only one that differed from the FU treatment for plant height, with a reduction of 14.58%. This lower plant height can be justified by the 55% reduction in soil available water compared to the limit required by papaya (f = 70%) (Fig. 6). Papaya growth is highly sensitive to soil water availability so that moderate water stress can substantially affect papaya growth^10,48^.

Papaya is considered a semi-woody giant herb; that is, it has a deficient anatomical support structure. Therefore, turgor pressure sustains the plant, and fruit weight significantly^48^. In this sense, slight differences in turgor pressure could induce different stem diameters. However, differences for stem diameters were not observed from 140 DAT (BA experiment) and 90 DAT (MG experiment). The highest stem diameter was observed in the treatments, PRD 14 50%, PRD 7 50%, and PRD 21 35% only at 80 DAT; however, significant differences were not observed among other treatments (Table 2). Similar results were reported by previous study^10^, who found no significant difference for stem diameter between papaya cultivated under the PRD methodology (30% WID reduction) and the control treatment (100% WID). Thus, stem diameter seems not to be a sensitive parameter to the interval of shifting of the irrigated side, even with a 50% reduction in WID at the flowering and production stages, which are the ones that require the largest water demand.

The larger leaf areas occurred in the treatments FU, PRD 7 (50 and 35%), and FX 35%. These larger leaf areas in the treatments are directly related to the higher soil water availability content in the soil of these treatments than the others; the higher the soil water content, the larger the papaya leaf area^9^.

PRD technique did not reduce the papaya roots growth. The root system's effective depth, that is, the depth corresponding to 80% of the total length of roots, varied between 0.42 m (PRD 21 50%) and 0.44 m (PRD 7 50%, PRD 14 50%, and FU). These values are very close to those (0.45 m) reported by previous study^49^ on the distribution of the papaya roots cv ‘Tainung nº 1’ under irrigation without water stress. Despite the absence of significant difference among treatments for both effective depth and an effective horizontal distance of the root system, in absolute values, there was a small reduction (2 cm) for both variables in papaya plants subjected to irrigated side alternation every 21 days and 50% reduction in WID. Such reduction indicates that the 85% decrease in soil available water (Fig. 5) on the side subjected to drying for 21 days causes a slight reduction of root growth in the soil profile, which did not occur in the treatments with alternation every 7 and 14 days. This reduction is due to the time over which roots are subjected to the condition of soil with low water availability (15%), which consequently increases its mechanical resistance, thus limiting root growth. A similar result was observed by previous study^17^, studying the distribution of the roots of ‘BRS Princesa’ banana cultivated under the PRD technique in the semi-arid region of Northern Minas Gerais.

In practical terms, studying the distribution of the roots of a crop is extremely important to identify the zone of highest activity of the root system, which in turn will assist in the calculation of the irrigation depth to be applied to the crop and in better definition of sensor position for soil water monitoring. The effective distance and effective depth of soil water extraction by papaya are like the values of the effective horizontal distance and effective depth of the root system^50^. Therefore, in the present study, the largest soil water extraction by the roots occurred within the root-zone bounded by the distance of the plant along the row or lateral drip line and soil depth of 0.44 m.

### Alternation of frequency of the irrigated side and NDVI

The months of most extreme thermal stress (i.e., from July to October, 2016), the NDVI varied on average from 0.46 to 0.62, with the highest values in the treatments with alternation frequency of the irrigated side of 7, 14, and 21 days, while the lowest values occurred in the FX and FU treatments (Fig. 8). This reduction was caused by the increase of VPD (Table 1) in August (3.2 kPa) and September (3.0 kPa), which increased the transpiration demand. Thus, to reduce water losses by transpiration, the papaya crop intensified the abscission and senescence of basal leaves, reducing the leaf area during these months.

Under the most thermal stress conditions, i.e., elevated VPD values, papaya plants substantially reduced the leaf area through basal leaf abscission, resulting in lower NDVI values. Also, plants submitted to the PRD technique during the period of greatest thermal stress displayed adaptability since the NDVI value did not decrease in the same intensity that occurred for the plants submitted to both FX and FU treatments (Table 2).

Before the greatest thermal stress period, the plants submitted to the PRD technique were in a condition of partial soil water limitation, which favored a control on the leaf area growth and loss of water through transpiration. At the same time, FU treatments had their leaf area fully developed. However, with the increases in VPD values, the rate of water absorption by roots is lower than the loss by transpiration; thus, these plants accelerate the leaf abscission to reduce the water loss by transpiration, reflected in lower NDVI in that period. The highest NDVI values in the BA than the MG experiment, mainly from 120 to 240 DAT (Fig. 9), reveals a greater vigor of the plants in the former, which justifies their greater yield (Table 4).

### Impacts of PRD on fruit yield and quality and crop water productivity

The reduction of the yield of treatments submitted to 50%WID in the MG experiment compared to the BA experiment was observed. Such a decrease of yield is justified by the severe attack of mites (*Tetranychus urticae*) along with the vegetative and reproductive development of papaya plants. Despite this reduction, the yield in all treatments was above the average yield of the country (39,892 kg ha^−1^) and Minas Gerais (32,360 kg ha^−1^), according to the study conducted by the Brazilian Institute of Geography and Statistics^24^. Besides the severe attack of mites in the MG experiment, these higher yields of BA experiment can be attributed to the higher water holding capacity of their soil and the lower rate of moisture reduction over time. Consequently, the plants from the BA experiment suffer lower water stress concerning the changes in the irrigated side of the plant row, which does not occur in the MG experiment (Figs. 5 and 6).

This lower yield is attributed to the 55% reduction in soil available water (PRD 21 50%) concerning allowable depletion for by papaya (30%), on the side subjected to drying (Fig. 5), which in turn caused leaf area reductions of 41%, 27%, and 16%, at 80, 140 and 240 DAT, respectively, compared to the FU treatment. This situation negatively impacted the photosynthetically active area of papaya. Papaya yield depends, among other things, on a light interception by leaves, since each leaf sustains 3 up to 4 fruits for cultivars from the Formosa groug^48^. According to previous study^51^, a reduction of 33% in the water supply to ‘UENF/Caliman 01’ papaya can cause a mean decrease of 51% in its marketable yield. The longer period of soil drying at available water levels below 50% influenced the absorption of water necessary for the adequate growth of the crop for yield. Papaya cultivation under conditions of water stress and high temperatures, as occurred in the present study, may reduce biomass production^10^ and net carbon assimilation^9,10^. These situations, therefore, negatively affect the development and yield of the crop.

In the treatments, PRD 7 35% and PRD 14 35%, the yields were 20 and 15% higher than those in the FX 35% treatment. This result demonstrates the superiority of plants subjected to the PRD technique compared to the irrigation in only one side of the plant (Table 4). Such superiority of the plants subjected to the PRD technique may be related to the increase in the concentration of abscisic acid in the leaves due to the soil's water deficit on the side subjected to drying^14^. An increase in leaf abscisic acid content induces partial stomatal closure, thus, contributing to the preservation of the leaf relative water content^52^ within a range in which the photosynthetic capacity and yield are not negatively affected^48^ and finally maintaining the production of photoassimilates at acceptable levels. Compared to the FU control treatment, the lower yield reductions occurred in the treatments, PRD 7 35% (6.53%), and PRD 14 35% (12.22%). Thus, the most recommended method for ‘Tainung nº 1’ papaya under semi-arid conditions is the alternation of the irrigated side every 7 days, followed by the alternation every 14 days, with a 35% reduction in WID.

Despite these reductions in irrigation depth applied, the average yields achieved in the PRD 50% treatments (7, 14, and 21 days) were higher than the average national yield by 43.46%, 43.21%, and 40.98% in the BA experiment and 34.02%, 34.54% and 26.39% in the MG experiment, respectively. In the PRD 35% treatments (7, 14, and 21 days), the yields in the BA experiment were 55.64%, 52.76%, and 44.18% higher, respectively. Therefore, these results, associated with the reduction in the irrigation depths applied, indicate how much the irrigation management strategies can maximize the rational use of water resources in irrigated fruit production, especially in areas with low rainfall regimes and high temperatures, as is the case of the Brazilian semi-arid region.

Except for the treatment PRD 14 50%, the mean fruit weights in the BA experiment were higher than those reported by previous study^53^, who found fruit weights for the ‘Tainung nº 1’ variety ranging from 900 to 1,100 g. In the MG experiment, the mean fruit weights are within this range (Table 4). Both in the BA and the MG experiments, the mean fruit weights in all treatments are within the range required by the market, which varies from 750 g to 2,400 g^54^.

Regarding the fruit quality, there was no statistical difference (p > 0.05) among treatments for titratable acidity (BA experiment) and total soluble solids (MG experiment), while PRD 7 50%, PRD 7 35%, FX 35%, and FU treatments resulted in greater average of total soluble solids (SS) in BA experiment. The average SS of both experiments is in the range of 9–14%, as found by previous study^55^ in each study with different cultivars of the “Formosa” group. The average SS values of all treatments of both experiments were larger than the ones presented by previous study^19^. These authors have grown papaya with water irrigation depths of 80%, 60%, and 50% ETc by using one or two emitters per plant. This superiority of SS is possibly related to the number of emitters per plant. The number of emitters influences the wetted area. Larger wetted areas favor water and nutrient uptake and assimilate production.

The SS/TA ratio represents the balance between sugars and acids in the fruits, which directly contributes to fruit aroma and flavor^56^. In general, the higher the SS/TA ratio, the lower the acidity of the fruit, which will have a more pronounced sweetness. Consequently, there will be greater acceptance by the consumer market. In the present study, there was no significant difference between treatments for the SS/TA ratio of fruits in any of the two experiments (Table 4). It is important to emphasize that full irrigation resulted in the highest SS and SS/TA ratio values in both experiments, with a significant effect for SS in the BA experiment. These results contrast with those observed by previous study^57^. They obtained the highest contents of soluble solids in 208 ‘UENF/Caliman 01’ papaya fruits when produced under lower irrigation depths (50% and 75% ETo).

Treatments PRD 21 35%, IF 35% e FU carried in BA showed smaller means of crop water productivity (CWP) than the others. The larger differences of CWP among PRD and FU treatments concerned PRD 7 50%, PRD 14 50%, and PRD 7 35%, with 2.94 kg/m^3^, 2.90 kg/m^3^, and 2.76 kg/m^3^, respectively (Table 4). These larger differences are directly related to the highest frequency of alternation of the irrigated side of crop row (7 and 14 days) associated with a larger WID reduction. The smallest interval between drying and wetting of soil root zone at both sides of crop row provided the smaller reduction of soil water availability at both sides (Fig. 5) and smaller difference of yields among these treatments and the one with full irrigation. This smaller difference in yields enhanced the CWP of the papaya crop positively. On the other hand, the smallest frequency of alternating the irrigated side of crop row (21 days) associated with the smallest reduction of irrigation water depth (35%) did not contribute to providing yield at amount enough to obtain CWP statistically larger than the one of FU treatment.

The CWP was significantly higher for treatments submitted to a 50% reduction of irrigation water depths than CWP from FU treatment of the MG experiment. These differences ranged from 1.59 to 2.39 kg of fruits produced by one cubic meter of water used by the papaya crop (Table 4). These results agree with the ones showed by previous study^6^, who worked with PRD treatments with a reduction of 50% WID on the banana crop, cv. BRS Princesa under the same soil and climate conditions.

Overall, our finding partially confirmed our initial hypothesis, i.e., increasing the frequency of shifting plant-row side irrigation increases the water availability, thereby increasing the some plant growth traits fruit yield and water productivity, however, did not significantly reduce total root length and effective depth and effective horizontal distance of papaya's root system. Additionally, increasing the frequency of shifting plant-row side irrigation did not compromise the titratable acidity, soluble solids, and SS/AT ratio of papaya fruits. At the same time, the irrigation water depth reduction of 50 and 35% under PRD enhances crop water productivity for papaya, except for PRD 21 35%. Moreover, PRD technique may save 35% of WID with alternation of the irrigated side of crop row every 7 days under a scarcity of the water resources. Finally, the NDVI was particularly important to compare the vegetation vigor between the experimental areas. We also demonstrated that NDVI was a useful tool for monitoring the papaya leaf area, especially when this parameter is altered by stressful conditions such as elevated air VPD.

## Conclusions

Soil water available reduced from the upper limit (field capacity) to levels of 44%, 50%, and 85% shifting plant-row irrigated side every 7, 14, and 21 days, respectively. PRD treatments did not promote significant modification in total root length and effective depth and effective horizontal distance; however, overall, the leaf area was substantially affected by PRD treatments, which reflected reduced NDVI values, especially in the MG experiment. However, such accentuated leaf abscission did not cause a significant reduction in papaya yield, as well as in papaya fruit quality traits (titratable acidity, soluble solids, and SS/AT ratio). Except for PRD 21 35%, the irrigation water depth reduction of 50 and 35% under PRD enhances water productivity for papaya. Under conditions of scarcity of water resources, the technique of partial drying with alternation of the irrigated side every 7 days and a 35% reduction in WID is the most recommended for papaya. The NDVI was particularly important to compare the vegetation vigor between the experimental areas. We also confirmed the potential of NDVI to monitor crops like papaya since we could notice its sensibility to identify when the crop is stressed, like the event with higher VPD in October 2016 and January 2017 in Bom Jesus da Lapa-BA.

## References

[1] Abdallah NA (2014). The impact of possible climate changes on developing countries. The needs for plants tolerant to abiotic stresses. GM Crops Food.

[2] Diffenbaugha NS, Burkea M (2019). Global warming has increased global economic inequality. Proc. Natl. Acad. Sci. USA.

[3] ANA (Agência Nacional de Águas). Atlas irrigação: Uso da água na agricultura irrigada, Brasília, DF, Brasil. https://www.ana.gov.br (2017)

[4] Meier J, Zabel F, Mauser W (2018). A global approach to estimate irrigated areas - A comparison between different data and statistics. Hydrol. Earth Syst. Sci..

[5] Moraes MMGA (2018). The impact of global change on economic values of water for Public Irrigation Schemes at the São Francisco River Basin in Brazil. Reg. Environ. Change.

[6] Coelho EF (2019). Soil-water-plant relationship and fruit yield under partial root-zone drying irrigation on banana crop. Sci. Agric..

[7] ANA (Agência Nacional de Águas). Conjuntura dos Recursos Hídricos no Brasil. Brasília, DF, Brasil. https://www.ana.gov.br (2015).

[8] Fernández JE (2020). Water use indicators and economic analysis for on-farm irrigation decision: A case study of a super high density olive tree orchard. Agric. Water Manag..

[9] Lima RSN (2016). Linking thermal imaging to physiological indicators in *Carica papaya* L. under different watering regimes. Agric. Water Manag..

[10] Lima RSN (2015). Partial rootzone drying (PRD) and regulated deficit irrigation (RDI) effects on stomatal conductance, growth, photosynthetic capacity, and water-use efficiency of papaya. Sci. Hortic..

[11] Lamaoui M (2018). Morphological, physiological, and biochemical responses to water stress in melon (*Cucumis melo*) subjected to regulated deficit irrigation (RDI) and partial rootzone drying (PRD). J. Crop Sci. Biotech..

[12] Zhang J (2019). Effect of partial root-zone drying irrigation (PRD) on the gas exchange and antioxidant enzymatic activities in alfalfa. J. Soil Sci. Plant Nutr..

[13] Santos DL (2021). Partial root-zone drying in field-grown papaya: Gas exchange, yield, and water use efficiency. Agric. Water Manag..

[14] El-Sadek A (2014). Water use optimization based on the concept of partial root zone drying. Ain Shams Eng. J..

[15] Shahabian M, Samar SM, Emdad MR (2012). Response of orange trees to deficit irrigation strategies in the north of Iran. Arch. Agron. Soil Sci..

[16] Effects on water use and crop production characteristics (2017). Consoli, S. *et al.* 2017. Partial root-zone drying irrigation in orange orchards. Eur. J. Agron..

[17] Santos MR (2016). Irrigação lateralmente alternada em lima ácida 'Tahiti' na região norte de Minas Gerais. Irriga.

[18] Çolak YB, Yazar A (2017). Evaluation of crop water stress index on Royal table grape variety under partial root drying and conventional deficit irrigation regimes in the Mediterranean Region. Sci. Hortic..

[19] Manjunath BL (2017). Partial root zone drying irrigation in papaya (*Carica papaya* L.) for enhanced water use efficiency under limited water situations. J. Hortl. Sci..

[20] Castricini A (2019). Quality of ‘Tainung 1’papaya produced by partial root zone drying. Rev. Bras. Frutic..

[21] Araus JL (2018). Translating high-throughput phenotyping into genetic gain. Trends Plant Sci..

[22] Congcong L (2019). Using NDVI percentiles to monitor real-time crop growth. Comput. Electron. Agric..

[23] Maselli F (2020). An improved NDVI-based method to predict actual evapotranspiration of irrigated grasses and crops. Agric. Water Manag..

[24] IBGE (Instituto Brasileiro de Geografia e Estatística). Sidra: Produção Agrícola Municipal. Brasília, DF, Brasil. https://sidra.ibge.gov.br (2019).

[25] QGIS Development Team. QGIS Geographic Information System. Open Source Geospatial Foundation, World (2017).

[26] Alvares CA (2013). Köppen’s climate classification map for Brazil. Meteor. Zeits..

[27] Santos HG (2013). Sistema brasileiro de classificação de solos.

[28] Teixeira, P. C. *et al.* Manual de métodos de análise de solo. https://www.infoteca.cnptia.embrapa.br/handle/doc/1085209 (Embrapa, Brasília, 2017).

[29] Arruda DM (2013). Phytogeographical patterns of dry forests sensu stricto in northern Minas Gerais State, Brazil. Ann. Acad. Bras. Cienc..

[30] Allen, R. G. *et al*. Crop evapotranspiration: guidelines for computing crop water requirements in *FAO Irrigation and Drainage Paper* No. 56, FAO, Rome, Italy (1998).

[31] Posse RP (2008). Evapotranspiração e coeficiente da cultura do mamoeiro. Eng. Agríc..

[32] Keller J, Bliesner RD (1990). Sprinkle and trickle irrigation.

[33] Mantovani EC, Bernardo S, Palaretti LF (2009). Irrigação Princípios e Métodos.

[34] Sanches, N.F. & Dantas, J.L.L. *O cultivo do mamão. Cruz das Almas*, Embrapa Mandioca e Fruticultura (Circular Técnica, 34) (1999).

[35] Alves, A.A.C. & Santos, E.L. Estimativa da área foliar do mamoeiro: método não destrutivo. In *Proceedings of the 17th Congresso Brasileiro de Fruticultura, Belém, PA*. CD-ROM (2002).

[36] Böhm W (1979). Methods of studying root systems.

[37] Kaspar TC, Ewing RP (1997). Rootedge: software for measuring root length from desktop scanner images. Agron. J..

[38] Morais PLD (2007). Pós-colheita de mamão híbrido UENF/Caliman 01 cultivado no Rio Grande do Norte. Rev. Bras. Frutic..

[39] Igbadun HE (2006). Crop water productivity of an irrigated maize crop in Mkoji sub-catchment of the Great Ruaha River Basin, Tanzania. Agric. Water Manag..

[40] AOAC (Association of Official Analytical Chemists). *Official methods of analysis* 15th ed. (Washington, 1990).

[41] Mandanici E, Bitelli G (2016). Preliminary comparison of Sentinel-2 and Landsat 8 imagery for a combined use. Remote Sens..

[42] Drusch M (2012). Sentinel-2: ESA’s optical high-resolution mission for GMES operational services. Remote Sens. Environ..

[43] Fernández-Manso A, Fernández-Manso O, Quintano C (2016). SENTINEL-2A red-edge spectral indices suitability for discriminating burn severity. Int. J. Appl. Earth Obs..

[44] Rouse, J. W. *et al.* Monitoring vegetation systems in the Great Plains with ERTS in *Third Earth Resources Technology Satellite–1 Syposium.* (eds Freden, S. C., Mercanti, E. P. & M. Becker, M.) 309–317. Volume I: Technical Presentations, NASA SP-351 (NASA, Washington, DC, 1974).

[45] Planet Labs Inc, Planet imagery and archive. https://www.planet.com/products/planet-imagery/ (2020).

[46] Cheng Y (2020). Phenology of short vegetation cycles in a Kenyan rangeland from PlanetScope and Sentinel-2. Remote Sens. Environ..

[47] Planet Team Planet Application Program Interface: In Space for Life on Earth. San Francisco, CA. https://api.planet.com (2017).

[48] Campostrini E, Tejero IFG, Zuazo VHD (2018). Environmental factors controlling carbon assimilation, growth, and yield of papaya (*Carica papaya* L.) under water-scarcity scenarios. Water Scarcity and Sustainable Agriculture in Semiarid Environment.

[49] Coelho EF, Santos MR, Coelho-Filho MA (2005). Distribuição de raízes de mamoeiro sob diferentes sistemas de irrigação localizada em latossolo de tabuleiros costeiros. Rev. Bras. Frutic..

[50] Carvalho GC, Coelho EF, Pamponet AJM (2014). Determinação do posicionamento de sensores de água do solo em mamoeiro irrigado por microaspersão e gotejamento. Rev. Magist..

[51] Posse RP (2009). Relação entre a produtividade do mamoeiro e o déficit hídrico (ky) na região Norte Fluminense. Rev. Bras. Eng. Agríc. Ambient..

[52] Pérez-Pérez JG (2018). Prolonged drying cycles stimulate ABA accumulation in *Citrus Macrophylla* seed lings exposed to partial rootzone drying. Agric. Water Manag..

[53] Costa AFS (2013). Botânica, melhoramento e variedades. Inf. Agropec..

[54] CEAGESP (Companhia de Entrepostos e Armazéns Gerais de São Paulo). São Paulo, SP, Brasil. http://www.ceagesp.gov.br (2019).

[55] Santana LRR, Matsuura FCAU, Cardoso RL (2004). Improved genotypes of papaya (Carica papaya L): sensory and physico-chemical evaluation. Food Sci. Technol..

[56] Viana ES (2015). Avaliação físico-química e sensorial de frutos de genótipos melhorados de mamoeiro. Pesq. Agropec. Trop..

[57] Silva MS (2013). Quality of papaya hybrid grown under different irrigation depths. Eng. Agríc..

